# Editorial: Functional near-infrared diffuse optical spectroscopy (fNIRS) to explore mental health

**DOI:** 10.3389/fpsyt.2022.1021622

**Published:** 2022-09-07

**Authors:** Yu Shang, Ting Li, Chong Huang

**Affiliations:** ^1^State Key Laboratory of Dynamic Measurement Technology, North University of China, Taiyuan, China; ^2^Institute of Biomedical Engineering, Chinese Academy of Medical Science and Peking Union Medical College, Tianjin, China; ^3^College of Engineering, University of Kentucky, Lexington, KY, United States

**Keywords:** fNIRS, cerebral cortex oxygenation, brain activation, schizophrenia, major depressive disorder, psychiatric diseases, anxiety and depression, verbal fluency task

Mental health disorders have been a long-term concern for society in general and have thus been increasingly studied by the scientific community. On the other hand, clinical assessment of mental health has heavily relied on subjective questionnaires and score sheets, due to a lack of objective measuring modalities. Over years, the functional near-infrared diffuse optical spectroscopy (fNIRS) has been attracting the attention of scientists and clinicians in the psychiatric field as a low cost and highly sensitive approach to assess cerebral hemodynamics that are closely associated with human functional capacities as well as symptoms of mental disorders.

This topic collection contains the review and research articles reporting the fNIRS methods for evaluating the mental health and probing various psychiatric diseases.The utilized methodologies & protocol as well as the key discoveries are summarized and highlighted in the following paragraphs.

## Reviews of fNIRS on mental disorders

In the article authored by Crum, the mental health and cognitive reconstruction of ecological neuroscience by fNIRS are extensively reviewed. The reviews reveal that the broad investigation of brain could be carried out by the fNIRS, which is helpful in design of the experimental tasks for mental health cultivation and cognitive interventions.

Suicide is the most severe tragedy due to long-term major depressive disorder (MDD). However, there are no established biomarker for predicting suicidality. In the article authored by Lee et al., a total of seven studies on use of fNIRS for assessment and prediction of suicidality are reviewed. In all the studies, the suicidal subjects demonstrated the reduced cerebral hemodynamic responses to the tasks, when compared with the healthy controls. Moreover, one of these studies also found a correlation between the fNIRS signals and severity of the suicidality. This review article implies the great value of fNIRS for predicting and preventing the tragedy of suicide.

The spontaneous brain activity at rest is a nature status in which the brain dysfunction might also be reflected, especially for the severe mental disorders such as schizophrenia. Yanagi et al. conducted a review on use of fNIRS to detect the cerebral malfunction in schizophrenia patients. The related studies emphasize the low frequency (0.01–0.08 Hz) of the cerebral hemodynamics, from which the parameter of the amplitude of low frequency fluctuations (ALFF), or fractional ALFF, were extracted. The review analysis shows that both parameters in the frontal cortex were decreased during the rest states in schizophrenia patients, indicating the consistent evidences of spontaneous brain activity for probing schizophrenia by fNIRS.

## Studies of fNIRS on diagnostics of psychiatric diseases

A large portion of the research articles contained in this topic collection are regarding the task protocol and the analysis approaches. In a study conducted by Ishii et al., a single-event related Japanese Shiritori task (i.e., word production) was applied to both MDD patients and healthy controls, and the fNIRS technology was used to evaluate the cerebral responses. During the period of word production, the MDD patients were found to have significantly smaller hemodynamic activation in prefrontal cortex area, and the oxy-hemoglobin changes were negatively correlated with the HAMD score. The outcomes presented in the study support the promise of combing the word production task and fNIRS for assessment of the cerebral function in MDD patients.

Verbal fluency task (VFT) is one of the most popular protocols to induce brain cortex activation, which is fully reflected in this topic collection. In the article authored by Wen et al., the VFT was compared with non-task (i.e., spontaneous brain activity), and the cerebral oxygenation activation on MDD, anxiety and depression (A&D) and healthy control (HC) populations were assessed by fNIRS. The outcomes show that VFT greatly enhanced the magnitude in power spectral analyses, but its intensity needs to be elevated for better characterizing the psychiatric diseases such as MDD and A&D.

In addition to VFT, other physiological manipulation are also emerging as alternative protocol to activate the brain cortex in psychiatric field. In this topic collection, Xiang et al. applied the Tower of London (TOL, i.e., the counting of picture movement) to the MDD and schizophrenia patients and compared with the healthy controls. During the performance of both protocols, the fNIRS successfully probed the difference in cerebral hemodynamics among the three groups, indicating the potential of TOL for distinguishing the psychiatric diseases from the healthy population. In another study conducted by Lang et al., the VFT protocol was compared with the high-level cognition task (HCT, in which the logical categorization is involved), and the prefrontal cortex oxygenation were measured on A&D patients and healthy controls. The results exhibit that more oxygen increment were activated by HCT than VFT, hence verifying it as an competitive protocol for A&D detection. Additionally, Tung et al. investigated the brain network and power during VFT task, and the fNIRS was used to assess the brain activation. The outcomes reveal that the high-functioning and low-functioning participants are different in activating the brain region connectivity, and the method of network topology appears to perform better than the method of activation power in analyzing the fNIRS data. This study also indicates that the left frontotemporal is a key region that actively responds to the VFT task.

In the above studies, the single parameter (e.g., average value, integral value, reaction time) extracted from oxygenation data were usually adopted to evaluate the brain activation, which may not be sufficient to differentiate among various disordered mental and healthy one. In recent year, several advanced data analysis approaches have been developed to improve the diagnostic accuracy for psychiatric diseases. As one of the representative examples, Chou et al. made the first attempt to utilize the two machine learning approaches, i.e., support vector machine (SVM) and deep neural network (DNN), to classify the first-episode schizophrenia (FES) patients and healthy subjects who have received the fNIRS during VTF tasks. The integral value and centroid value were used as the feature parameters for the machine learning. Both approaches were found to perform well in classifying the FES from healthy controls, with the satisfactory accuracy, sensitivity, and specificity. This study supports the use of artificial intelligence methods for screening of the psychiatric diseases.

## Studies of fNIRS on therapeutic evaluation of psychiatric diseases

Methylphenidate (MPH) is a widely-used medicine to treat the children with attention deficit hyperactivity disorder (ADHD) or minor brain dysfunction. Thus far, the standard behavior approach to evaluate the MPH's effects have not been well established. Jang et al. used virtual reality working memory task to stimulate the children with ADHD who also received the MPH treatment, and fNIRS was used to assess the cerebral oxygenation changes throughout the protocol. The outcomes show that reaction time of oxygen data was shorten after MPH treatment, indicating the potential of fNIRS in assessment of the therapeutic effects.

Acupuncture is a physical modality to treat a variety of mental disorders. Nevertheless, there are no objective criteria to evaluate the treatment effects. In a study conducted by Zhang et al., the VFT task was applied to the MDD patients after they received the acupuncture treatment and measured by fNIRS. Based on the data of cerebral hemodynamics, a single session of acupuncture did not show to improve the brain activation with the patients with mild and moderate depression. By contrast, the significant improvement of brain activation was observed in severe depression patients, and the degree of activation is correlated with the HAMD score. This study demonstrates the possibility of using fNIRS for therapeutic evaluation of mental disorders. Similarly, in another study on treatment of depression patient with repetitive transcranial magnetic stimulation (rTMS) (Kawabata et al.), the cerebral hemodynamic enhancement were also observed, indicating the great promising of fNRS for timely evaluation of therapy effects.

We hope this topic collection will provide timely and sufficient information for the scientists and clinicians working in field of psychiatry, particularly for those with fNIRS applications. We believe the emerging fNIRS technologies, along with the advanced brain activation protocol and data analysis approaches, will be great beneficial for diagnosis and therapy of various psychiatric diseases.

## Author contributions

YS wrote the draft of editorial article. CH and TL revised the content and involved in the content writing discussion. All authors contributed to the article and approved the submitted version.

## Conflict of interest

The authors declare that the research was conducted in the absence of any commercial or financial relationships that could be construed as a potential conflict of interest.

## Publisher's note

All claims expressed in this article are solely those of the authors and do not necessarily represent those of their affiliated organizations, or those of the publisher, the editors and the reviewers. Any product that may be evaluated in this article, or claim that may be made by its manufacturer, is not guaranteed or endorsed by the publisher.

